# A kinetic model of phospholipase C-γ1 linking structure-based insights to dynamics of enzyme autoinhibition and activation

**DOI:** 10.1016/j.jbc.2022.101886

**Published:** 2022-03-31

**Authors:** Jamie L. Nosbisch, James E. Bear, Jason M. Haugh

**Affiliations:** 1 Biomathematics Graduate Program, North Carolina State University, Raleigh, North Carolina, USA; 2 Department of Cell Biology and Physiology, UNC Lineberger Comprehensive Cancer Center, University of North Carolina School of Medicine, Chapel Hill, North Carolina, USA; 3 Department of Chemical and Biomolecular Engineering, North Carolina State University, Raleigh, North Carolina, USA

**Keywords:** phospholipase C, mathematical model, phosphoinositide, protein kinase C, receptor tyrosine kinase, cSH2, C-terminal SH2, DAG, diacylglycerol, MARCKS, myristoylated alanine-rich C-kinase substrate, nSH2, N-terminal SH2, PA, phosphatidic acid, PFL, positive feedback loop, PH, Pleckstrin homology, PIP2, phosphatidylinositol-4,5-bisphosphate, PLC, phospholipase C, PLC-γ1, phospholipase C-γ1, RTK, receptor tyrosine kinase, SH2, Src homology 2, SH3, Src homology 3

## Abstract

Phospholipase C-γ1 (PLC-γ1) is a receptor-proximal enzyme that promotes signal transduction through PKC in mammalian cells. Because of the complexity of PLC-γ1 regulation, a two-state (inactive/active) model does not account for the intricacy of activation and inactivation steps at the plasma membrane. Here, we introduce a structure-based kinetic model of PLC-γ1, considering interactions of its regulatory Src homology 2 (SH2) domains and perturbation of those dynamics upon phosphorylation of Tyr783, a hallmark of activation. For PLC-γ1 phosphorylation to dramatically enhance enzyme activation as observed, we found that high intramolecular affinity of the C-terminal SH2 (cSH2) domain–pTyr783 interaction is critical, but this affinity need not outcompete the autoinhibitory interaction of the cSH2 domain. Under conditions for which steady-state PLC-γ1 activity is sensitive to the rate of Tyr783 phosphorylation, maintenance of the active state is surprisingly insensitive to the phosphorylation rate, since pTyr783 is well protected by the cSH2 domain while the enzyme is active. In contrast, maintenance of enzyme activity is sensitive to the rate of PLC-γ1 membrane (re)binding. Accordingly, we found that hypothetical PLC-γ1 mutations that either weaken autoinhibition or strengthen membrane binding influence the activation kinetics differently, which could inform the characterization of oncogenic variants. Finally, we used this newly informed kinetic scheme to refine a spatial model of PLC/PKC polarization during chemotaxis. The refined model showed improved stability of the polarized pattern while corroborating previous qualitative predictions. As demonstrated here for PLC-γ1, this approach may be adapted to model the dynamics of other receptor- and membrane-proximal enzymes.

Chemotaxis, the process by which cells direct their migration based on a soluble chemical cue, is a critical process for embryonic development, the immune response, and wound healing (1). Fibroblasts and other mesenchymal cells respond chemotactically to platelet-derived growth factor through a phospholipase C (PLC)/PKC signaling pathway (2). Aberrant activation of PLC-γ1, the specific isozyme of PLC involved in this pathway, has been implicated in a number of disease states, including immune disorders and cancer metastasis (3, 4). Recent work revealing the structure of PLC-γ1 has provided more detail into the multistep mechanism of PLC-γ1 activation from its basally autoinhibited state (5, 6). Therefore, it is important to connect the details of PLC-γ1 structural dynamics to its signaling function, which is also likely to inform the understanding of related signaling enzymes. Understanding the mechanism of PLC-γ1 activation will, in turn, inform our understanding of chemotactic gradient sensing.

To date, 13 mammalian isozymes of PLC, classified in six different structural families, have been identified. Common to all PLC isozymes is their enzymatic function: calcium-dependent hydrolysis of phosphatidylinositol-4,5-bisphosphate (PIP_2_) to form diacylglycerol (DAG) and inositol-1,4,5-triphosphate (7). This activity is basally autoinhibited by an X–Y linker that separates the X- and Y-boxes of the catalytic core. For the PLC-γ1 isozyme, this X–Y linker region contains a split Pleckstrin homology (PH) domain, two Src homology 2 (SH2) domains, and an Src homology 3 (SH3) domain. In the absence of stimulation, these X–Y linker domains sit atop the catalytic core and prevent the core from interacting with lipids at the plasma membrane (8). PLC-γ1 activation is mediated by receptor tyrosine kinases (RTKs), G protein–coupled receptors, cytokine receptors, and T-cell receptors (9). When activating signals are present, PLC-γ1 is phosphorylated, leading to a structural rearrangement that shifts the X–Y linker off the catalytic core, allowing the enzyme to bind to the membrane and hydrolyze PIP_2_ (6, 8).

In this work, we developed a rule- and structure-based model to study the kinetics of PLC-γ1 activation. In rule-based modeling, molecular interactions are specified by reaction rules that, when applied to molecules and their constituent domains/motifs, generate a reaction network. The main advantage of rule-based modeling is that each rule can be applied to a particular molecule in a variety of states permitted by the rule, and thus, the combinatorial complexity of signaling networks is accommodated (10). Using this approach, we analyzed the effects of particular mechanistic steps on the kinetics and steady-state level of PLC-γ1 activation. This approach elucidated how known gain-of-function PLC-γ1 mutations/variants, which have been implicated in cancer and other disease states, function (5, 6). In the context of the model, we considered mutations that disrupt autoinhibition of the catalytic core by the C-terminal SH2 (cSH2) domain (*K*_*c*_ variants) and mutations that increase the affinity of the catalytic core binding to the membrane (*K*_*a*_ variants). By varying the appropriate reaction rates for each mechanism, we predicted how steady-state enzymatic activity is increased. And, at the level of activation kinetics, a clear distinction between *K*_*c*_ and *K*_*a*_ variants emerged; *K*_*c*_ mutations confer faster transition from the basal to the active state of PLC-γ1, whereas *K*_*a*_ mutations confer slower reversion of the active state. This insight could inform mechanistic characterization of PLC-γ1 variants in terms of priming *versus* maintenance of enzymatic activity.

We also applied the structure-based model of PLC-γ1 activation to the mechanistic understanding of PLC/PKC signaling during chemotactic gradient sensing, building upon previous models (11, 12). We substituted the present PLC-γ1 activation network to refine our recent model of PLC/PKC polarization, which incorporated two positive feedback loops (PFLs) involving the lipid intermediate, phosphatidic acid (PA) (12). Integrating the structure-based model and a modified version of one of the PFLs, in which the affinity of catalytic core binding to the membrane (*K*_*a*_) is enhanced by PA, we found that this extended model enhanced previous model predictions regarding the spatial polarization of the signaling circuit. Considering the aforementioned gain-of-function changes (*K*_*c*_ and *K*_*a*_ variants), we predict that increased enzymatic activity is accompanied by diminished ability to polarize the signaling pathway.

## Results

### A structure-based model of PLC-γ1 regulation

**Figure 1:**
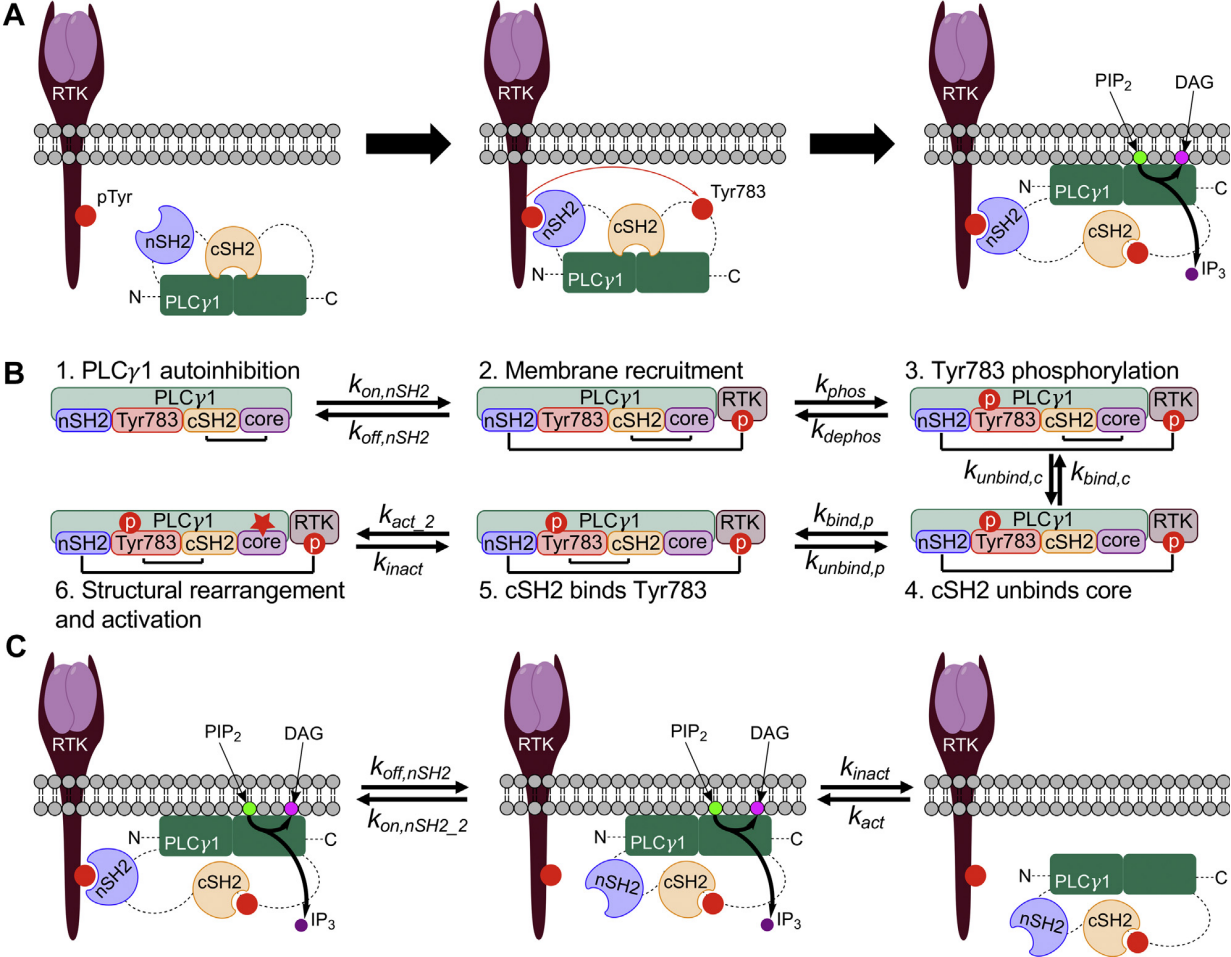
**Multistep activation and inactivation of PLC-γ1.***A*, illustration of PLC-γ1 activation steps. The catalytic core, separated into X- and Y-boxes, is depicted in *green*, with the regulatory X–Y linker region containing the nSH2 and cSH2 domains on *top*; N and C termini are also shown. PLC-γ1 binds to, and is phosphorylated on Tyr783 by, an activated autophosphorylated RTK. *B*, depiction of transitions between interaction and phosphorylation states of PLC-γ1 in the canonical activation sequence, with corresponding rate constants. *C*, illustration of a two-step inactivation sequence for PLC-γ1, where the nSH2–RTK interaction dissociates first, followed by the catalytic core–membrane interaction; the reverse order of these events presents another inactivation path. Rebinding events prolong the complete process and thus enhance the maintenance of PLC-γ1 recruitment and activity. cSH2, C-terminal SH2; nSH2, N-terminal SH2; PLC-γ1, phospholipase C-γ1; RTK, receptor tyrosine kinase.

We developed a model of PLC-γ1 to study its activation kinetics in light of recent structural data implicating a multistep activation process involving multiple domains and phosphorylation of Tyr783. The PLC-γ1 enzyme is basally autoinhibited by an X–Y linker region containing a number of regulatory domains (PH, SH2, and SH3 domains) that physically block the enzyme’s catalytic core, preventing it from interacting with lipids at the plasma membrane (autoinhibition). The canonical path for PLC-γ1 activation is depicted in Figure 1, *A* and *B* (see Fig. S1 for the full network of reactions). This series of priming steps starts with an active receptor complex, which has a phosphorylated tyrosine residue in its cytoplasmic tail. The N-terminal SH2 (nSH2) domain of the autoinhibited PLC-γ1 enzyme binds to the phosphorylated receptor, thus recruiting the inactive enzyme from the cytosol. While bound, Tyr783 of PLC-γ1, which is located between the cSH2 domain and the SH3 domain, may be phosphorylated. The cSH2 domain is usually in contact with a C2 domain next to the Y-box of the catalytic core, an interaction that confers the autoinhibition of PLC-γ1. When Tyr783 is phosphorylated, it serves as an intramolecular ligand that competes with the C2 domain for binding the cSH2 domain. For the cSH2 domain to bind pTyr783, a substantial rearrangement occurs, with the X–Y linker shifted away from the catalytic core. This exposes a hydrophobic ridge on the catalytic core, allowing the enzyme to insert itself into the membrane and exert catalytic activity (6).

This description of PLC-γ1 activation must be accompanied by a random two-step inactivation process for the enzyme to completely dissociate from the membrane (Fig. 1*C*). When the core is bound to the membrane and actively hydrolyzing PIP_2_, the nSH2 domain may dissociate from the RTK. While the core is independently bound to the membrane, it remains active. Given the close proximity of the enzyme to the receptor, the nSH2 domain has a chance to rebind the receptor. By the same token, while the nSH2 domain is bound to the receptor, the core may dissociate from the membrane and become inactive; however, it remains in close proximity to the membrane and has a chance to rebind. Only when the nSH2 domain unbinds the receptor and the core dislodges from the membrane will PLC-γ1 return to the cytosol. Thus, formulation of a thermodynamically complete regulation model yields the insight that rebinding events enhance the maintenance of enzyme activity at the membrane.

### Phosphorylated Tyr783 relieves autoinhibition of PLC-γ1 by intramolecular competition for the cSH2 domain, permitting activation even when the cSH2 domain greatly prefers to engage the catalytic core

**Figure 2:**
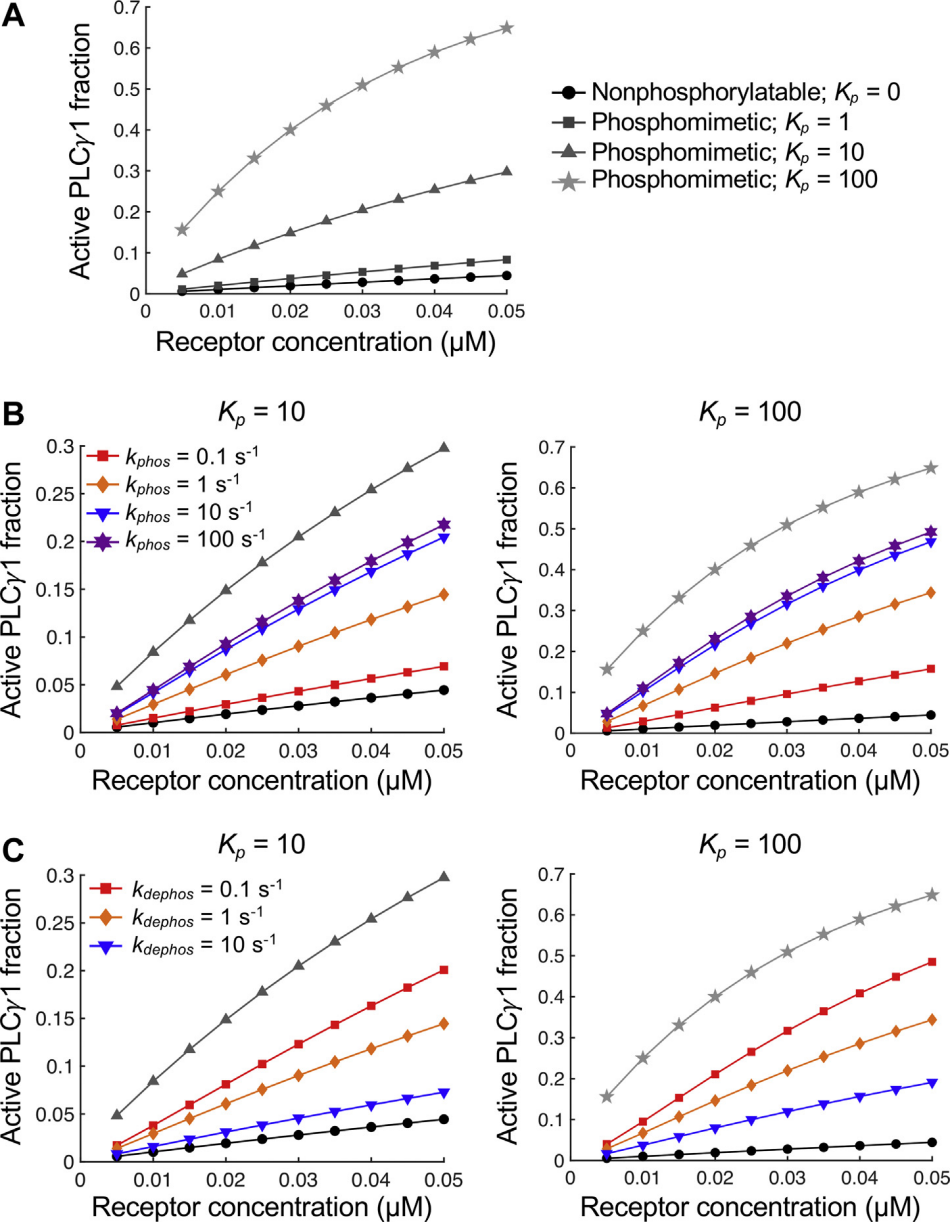
**Sensitivity of PLC-γ1 activation to Tyr783 phosphorylation and dephosphorylation rates.** The steady-state fraction of active PLC-γ1 is plotted as a function of the initial receptor concentration, with parameter variations as indicated. *A*, results for hypothetical nonphosphorylatable and phosphomimetic “mutants.” Note that the legend for this plot carries over to *B* and *C*. *B*, plots where the Tyr783 phosphorylation rate constant, *k*_*phos*_, was varied for *K*_*p*_ = 10 and *K*_*p*_ = 100. *C*, same as (*B*) but with variation of the Tyr783 dephosphorylation rate constant, *k*_*dephos*_. PLC-γ1, phospholipase C-γ1.

Considering the proposed role of Tyr783 phosphorylation in occupying cSH2 and thus relieving the autoinhibition of PLC-γ1, we explored how the rates of Tyr783 phosphorylation and dephosphorylation and the affinity of the cSH2 domain binding to phosphorylated Tyr783 (*K*_*p*_ = *k*_*bind,p*_*/k*_*unbind,p*_) affect PLC-γ1 activation (Fig. 2). In our model, it is assumed that Tyr783 can only be phosphorylated when the nSH2 domain is bound to the receptor, and it cannot be dephosphorylated while occupied by the cSH2 domain.

We first considered two extreme cases in which the PLC-γ1 enzyme was “mutated,” such that it is either nonphosphorylatable (Y783F is considered as such) or constitutively phosphorylated (phosphomimetic; we note that no such variant exists, because mutating Tyr to an acidic residue does not confer binding to SH2 domains). These served as limiting cases for PLC-γ1 activity, while variations were made to the phosphorylation and dephosphorylation rates to explore intermediate behavior. Our model description permits PLC-γ1 activation without phosphorylation of Tyr783, in the infrequent event that the cSH2 domain spontaneously unbinds the core, and the core binds to the membrane and becomes active before the autoinhibitory influence of cSH2 is re-established. This results in the low level of activation seen for the nonphosphorylatable case (Fig. 2*A*). For the phosphomimetic case, increasing the value of *k*_*bind,p*_ (and thus *K*_*p*_) results in progressively higher levels of PLC-γ1 activity for a given receptor input. A key prediction, based on analysis of the steady-state equations for this case, is that *K*_*p*_ >> 1 is required for Tyr783 phosphorylation to substantially influence PLC-γ1 activation, but the value of *K*_*p*_ need not be greater than or even comparable to *K*_*c*_, the affinity constant of the autoinhibition; indeed, the analysis shows that *K*_*c*_ must also be large (Text S1). With *K*_*p*_ = 10 and *K*_*c*_ = 100, the phosphomimetic “mutant” is substantially activated relative to the nonphosphorylatable one (Fig. 2*A*). To put it simply, even modest relief of a strong autoinhibition is effective relative to no relief.

Considering then the wildtype PLC-γ1, with varying rates of Tyr783 phosphorylation (Fig. 2*B*) or dephosphorylation (Fig. 2*C*), respectively, it is increasingly more or less likely for the cSH2 domain to be occupied by the Tyr783 site and not the core, leading to higher or lower levels of activation. Interestingly, the effect of increasing the phosphorylation rate apparently saturates (Fig. 2*B*; compare *k*_*phos*_ values of 10 and 100 s^−1^), achieving a maximum PLC-γ1 activity well below the level of the phosphomimetic case. The interpretation, supported by analysis of the model equations (Text S1), is as follows: even when phosphorylation of receptor-bound PLC-γ1 is rapid, cytosolic PLC-γ1 tends to be dephosphorylated; thus, the activation path involving direct binding of wildtype PLC-γ1 to the membrane is abrogated relative to the phosphomimetic case.

### Membrane association frequency, not phosphorylation frequency, affects the maintenance of active PLC-γ1 at the membrane

**Figure 3:**
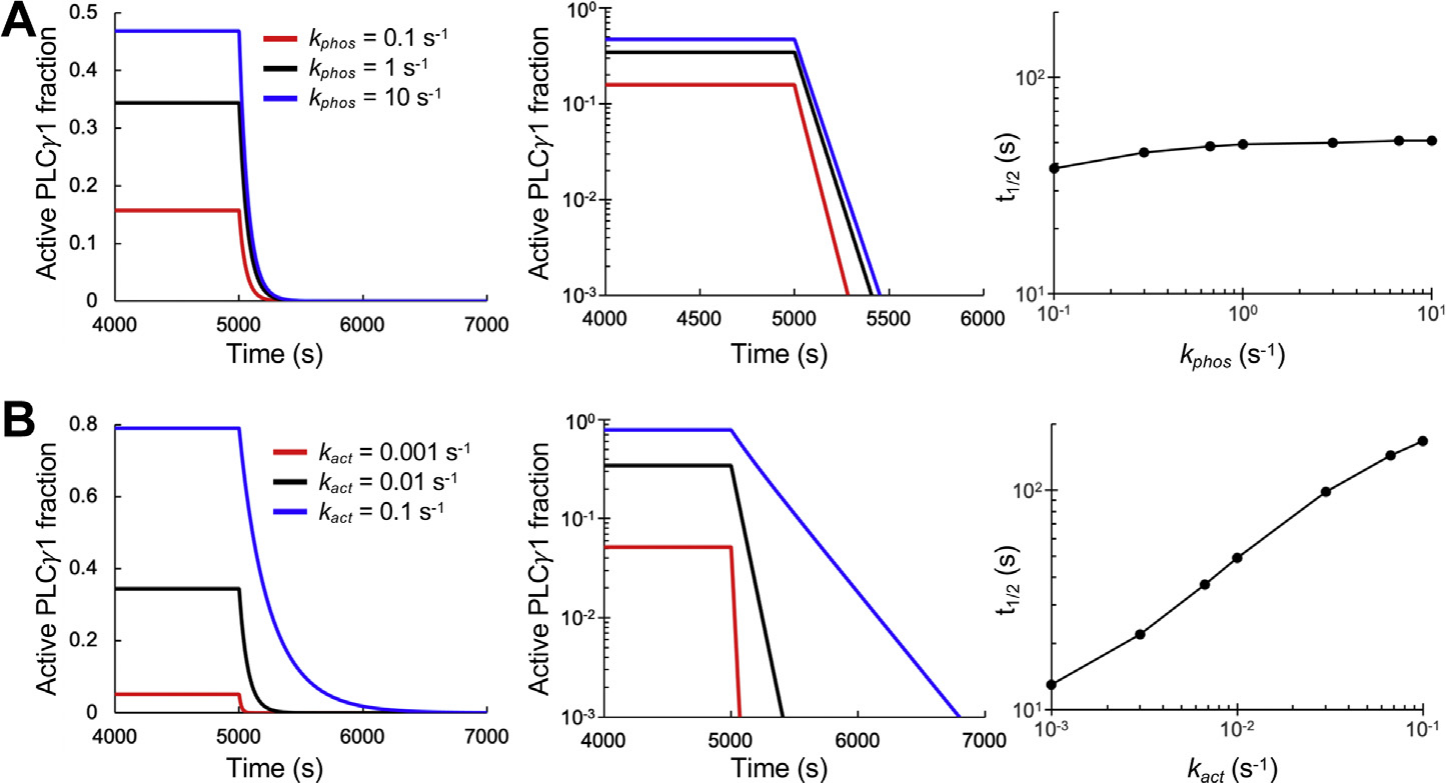
**Lifetime analysis reveals microscopic steps that affect maintenance of PLC-γ1 activity.** Time courses showing the decay of active PLC-γ1 species, with the rate constants for recruitment from the cytosol dropped to zero at 5000 s, are presented as linear and semilog plots, and the corresponding half-life (*t*_1/2_) values are also plotted. *A*, variation of the phosphorylation rate constant, *k*_*phos*_, reveals a modest effect on activity lifetime. *B*, variation of the activation rate constant, *k*_*act*_, reveals a sensitive effect on activity lifetime. PLC-γ1, phospholipase C-γ1.

Given the sensitivity of steady-state enzyme activity to the rate of PLC-γ1 phosphorylation, we asked whether or not this sensitivity translates to the lifetime of the enzyme at the membrane, another potential experimental readout. To test this, we ran simulations to steady state as usual and then switched off the reactions that allow PLC-γ1 to be recruited from the cytosol. Hence, we assessed the decay of the active species over time. The half-life for decay, calculated as the time required for the fraction of active PLC-γ1 to reach half its steady-state concentration, is relatively insensitive to *k*_*phos*_ despite 10-fold changes in either direction (Fig. 3*A*). Analysis of the model explains that, while PLC-γ1 is in an active state, phosphorylated Tyr783 tends to be occupied by the cSH2 domain and thus well protected from dephosphorylation. The need for Tyr783 phosphorylation to occur at an appreciable rate is prior to activation, just after recruitment from the cytosol.

The membrane association (activation) rate constant, *k*_*act*_, was analyzed in the same manner, with *k*_*act*_ (and *k*_*act*___*2*_ = χ*k*_*act*_) varied 10-fold in either direction relative to the base-case value. The time courses for the decay of active PLC-γ1 clearly show slower rates (increase in half-life) of decay with increasing *k*_*act*_ (Fig. 3*B*). This suggests that the ability of the enzyme to quickly rebind the membrane while it is still anchored to the receptor by the nSH2 domain is important for maintaining the activity of PLC-γ1. Changing certain other parameters, including the phosphorylation rate, does not have this effect. Taken together, the lifetime results suggest a distinction between microscopic rate steps that affect priming of PLC-γ1 activity and those that affect its maintenance.

### Hypothetical activating mutations affect both the magnitude and kinetics of PLC-γ1 activation in interpretable ways

A number of mutations in the PLC-γ1 enzyme have been implicated in various disease states and cancers, and *in vitro* activity assays have been used to confirm that such disease-linked substitutions or deletions possess higher PLC activity. Now that the structure of full-length PLC-γ1 has been reported, the mutations can be mapped to the structure of the protein. Although these mutations are distributed in a few different regions, most appear to overlap with the surfaces associated with autoinhibition of PLC-γ1 (5). Two possible mechanisms describing how these mutations are functioning include disruption of the cSH2 interaction with the core and enhancement of the affinity of the core for the membrane (6).

**Figure 4:**
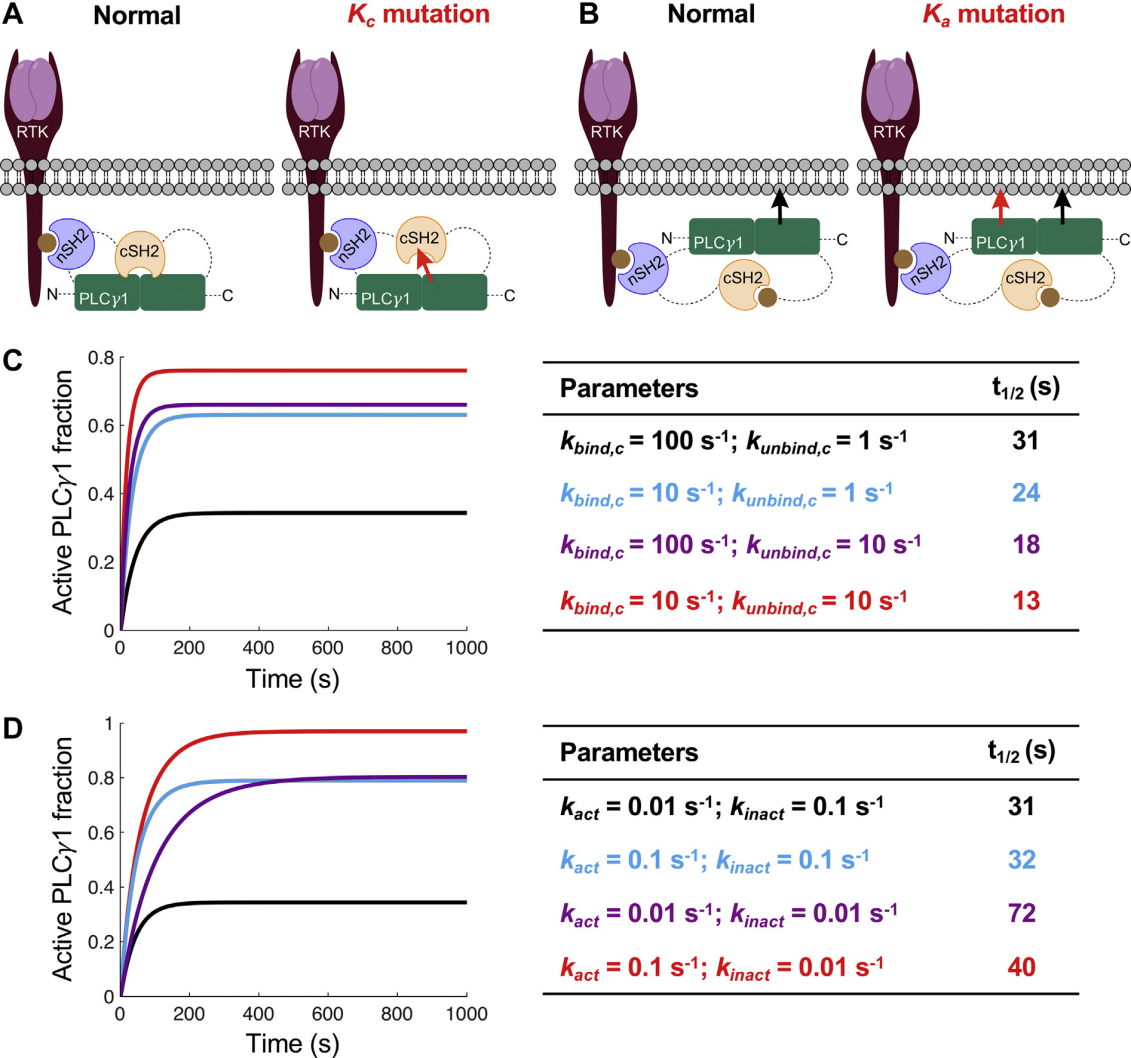
**Predicted effects of hypothetical mutations on PLC-γ1 activation kinetics.***A*, illustration of hypothetical *K*_*c*_ mutations that disrupt the autoinhibitory occupation of the catalytic core by the cSH2 domain. *B*, illustration of hypothetical *K*_*a*_ mutations that enhance binding of the catalytic core to the membrane. *C*, time courses of PLC-γ1 activation comparing wildtype (base-case) parameter values (*black*) to various *K*_*c*_ mutation scenarios. The corresponding *t*_1/2_ values are tabulated. *D*, same as (*C*), except for *K*_*a*_ mutation scenarios. cSH2, C-terminal SH2; PLC-γ1, phospholipase C-γ1.

Using our model, we mimicked the mechanisms attributed to these mutations by varying parameters associated with the cSH2 domain binding the core (*K*_*c*_ = *k*_*bind,c*_*/k*_*unbind,c*_) (Fig. 4*A*) and the uninhibited enzyme binding the membrane (*K*_*a*_ = *k*_*act*_*/k*_*inact*_) (Fig. 4*B*). As expected, we found that varying the *K*_*c*_ parameters, such that the cSH2 domain interaction with the core is hampered, results in increased PLC-γ1 activity, consistent with the *in vitro* assay results. Moreover, we observed that the time to reach half of the steady state is shorter (faster kinetics) for all the mutated *K*_*c*_ parameter sets (Fig. 4*C*). Varying the *K*_*a*_ parameters, such that the uninhibited enzyme has higher affinity for the membrane, also results in higher PLC-γ1 activity; however, examining activation kinetics revealed that increasing *K*_*a*_ yields slower activation kinetics (Fig. 4*D*), the opposite effect of reducing *K*_*c*_. Considering the spectrum of hypothetical variants, there is greater than fivefold difference in the *t*_1/2_ values when *K*_*p*_ = 100 and that difference increases to sevenfold when *K*_*p*_ = 10 (Fig. S2).

**Figure 5:**
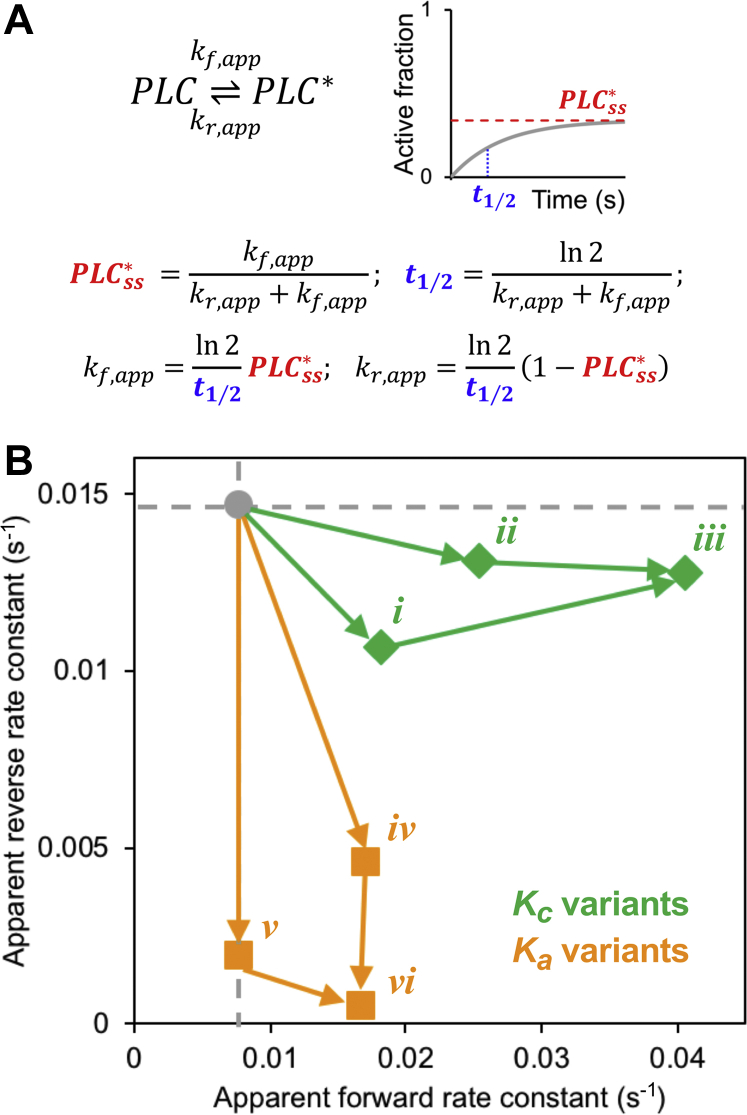
**Parsing effects of hypothetical mutations on priming and maintenance of PLC-γ1 activity.***A*, a reduced two-state model of enzyme activation is defined by apparent forward and reverse rate constants, which are estimated from the steady-state active fraction and *t*_1/2_ values of the activation time course. *B*, plot of the apparent forward and reverse rate constant values for *K*_*c*_ variants (*green diamonds*; [*i*] reduced *k*_*bind,c*_; [*ii*] increased *k*_*unbind,c*_; [*iii*] combination of [*i*] and [*ii*] and *K*_*a*_ variants (*orange squares*; [*iv*] increased *k*_*act*_; [*v*] decreased *k*_*inact*_; [*vi*] combination of [*iv*] and [*v*]) relative to the base case (*gray circle* and *dashed lines*). PLC-γ1, phospholipase C-γ1.

While this stark difference in activation kinetics could *per se* aid in distinguishing actual PLC-γ1 variants, one might wonder how to interpret a hypothetical experiment showing a *t*_1/2_ value that is hardly different from wildtype, as in the case of increasing the *k*_*act*_ parameter (Fig. 4*D*); however, the fact that the *t*_1/2_ value is approximately the same, yet the steady-state activity is dramatically increased, is significant. To discern this, we analyzed the activation kinetics predicted by the full model in terms of a reduced model that considers only two states of PLC-γ1, inactive and active (Fig. 5). The two parameters of the reduced model (apparent forward and reverse rate constants) are directly estimated from the *t*_1/2_ and steady-state fraction of PLC-γ1 activation (Fig. 5*A*). Applying this analysis to compare the predicted time courses of the *K*_*c*_ and *K*_*a*_ variants, we find that they cluster in a far more meaningful way. Whereas *K*_*c*_ mutations yield greater changes in promoting activation in the forward direction, *K*_*a*_ mutations yield greater changes in slowing deactivation in the reverse direction (Fig. 5*B*). Consistent with this interpretation, the lifetimes of the active state were quantified for the hypothetical variants, as in Figure 3, and we found that the half-life values are accurately predicted from the corresponding apparent reverse rate constant values (Fig. S3). Together, these results support the assertion that effects on priming and maintenance of PLC-γ1 activity have distinct kinetic signatures.

### Amplification of signaling in a combined model of PLC-γ1 activation and PLC/PKC polarization

Having modeled and analyzed the regulation of PLC-γ1 activity *via* its modular domain interactions, we sought to understand its implications for PLC/PKC signaling, specifically its function in chemotactic gradient sensing. Our previously published polarization model (12), which simplified PLC-γ1 activation as a single-step process, was refined to include the full network of 16 PLC-γ1 states. A key aspect of the polarization model is a PFL, PFL 1, by which the lifetime of active PLC-γ1 at the plasma membrane is prolonged by its interaction with PA, a lipid intermediate formed in the metabolism of DAG. In the new combined model, we recognized that PA likely enhances the membrane-binding/activation step; the analysis of active PLC-γ1 lifetime presented previously, showing that the lifetime is sensitive to the activation rate constant, *k*_*act*_ (Fig. 3*B*), was also encouraging. We refer to this modification of PFL 1 as PFL 1∗.

**Figure 6:**
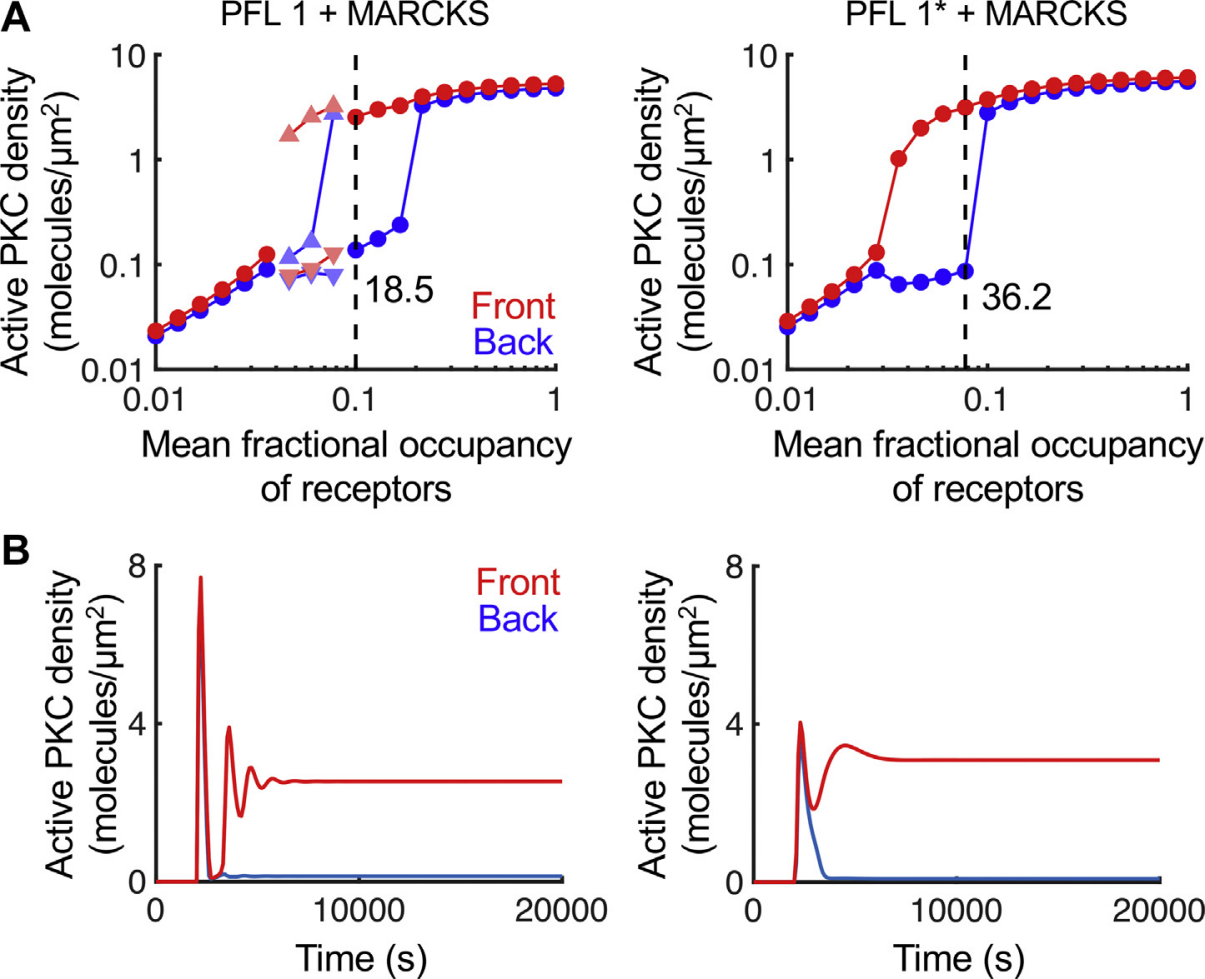
**Amplified gradient sensing in a combined model of PLC-γ1 activation and PLC/PKC polarization.***A*, in each input–output plot, the steady-state concentrations of active PKC at the front (*red circle*) and back (*blue circle*) of the cell are plotted as a function of the mean fractional occupancy of receptors, *rfrac*, with a constant 10% gradient steepness. When the simulations produced oscillations, the maxima (*triangles*) and minima (*inverted triangles*) of the oscillations are plotted. The simulation achieving steady state with the highest front/back ratio is denoted by the *dashed vertical line*. The two plots compare the results of the previously published model, with positive feedback loop 1 (PFL 1) and regulation of MARCKS (PFL 1 + MARCKS; *left*) and the corresponding simulations with the modified feedback loop (PFL 1∗ + MARCKS; *right*). *B*, time courses for the simulations with highest front/back ratio. MARCKS, myristoylated alanine-rich C-kinase substrate; PLC, phospholipase C; PLC-γ1, phospholipase C-γ1.

The combined model with PFL 1∗ and regulation of myristoylated alanine-rich C-kinase substrate (MARCKS) (but without PFL 2; refer to Ref. (12)) has distinct advantages relative to the corresponding case of the previous model (Fig. 6). At a relative gradient steepness of 10%, PFL 1∗ is able to synergize with MARCKS regulation to polarize the signaling pathway to a somewhat higher degree than the original model, and the combined model does not produce the oscillations observed for the original model for certain receptor occupancy levels (Fig. 6*A*). The time courses for the simulations showing the highest amplification also revealed differences, with the combined model showing simpler, and thus arguably more plausible, kinetics (Fig. 6*B*).

**Figure 7:**
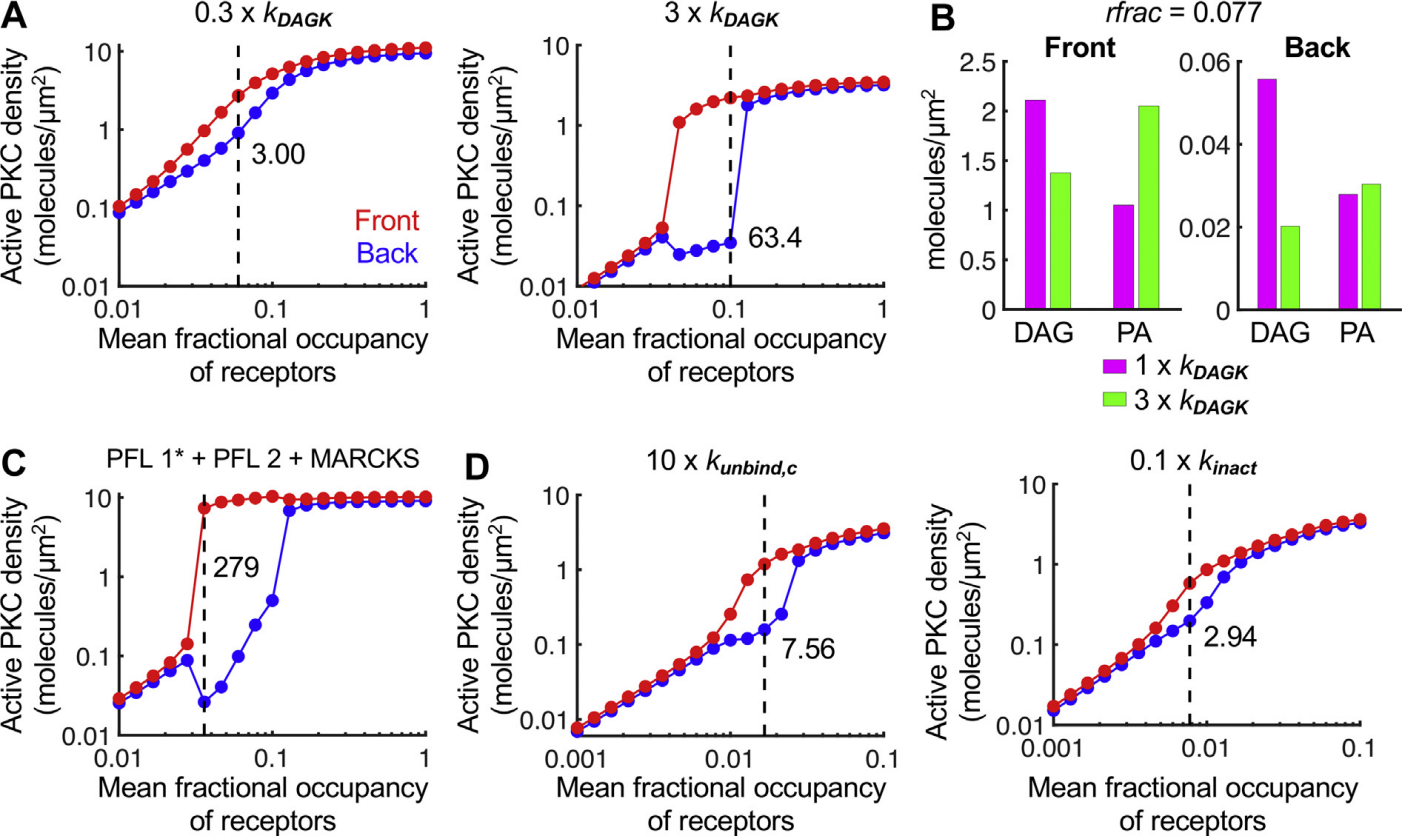
**Predicted effects of parameter variations on PLC/PKC polarization in the combined model.** The input–output plots shown in (*A*), (*C*), and (*D*) are formatted, and the maximum front/back ratios are shown, as for Figure 5*A*. *A*, relative to the base-case parameter set associated with the combined PLC/PKC polarization model (PFL 1∗ + MARCKS), the DAG phosphorylation rate constant, *k*_DAGK_, was taken at 0.3 or 3 times its base value. *B*, bar plots comparing the DAG and PA steady-state concentrations at the front and back of the cell for 1× and 3× *k*_DAGK_ simulations, with *rfrac* = 0.077. *C*, PFL 2 is added (PFL 1∗ + PFL 2 + MARCKS), with associated parameters, n = 2, *K*_*PLD*_ = 0.1, and γ∗*V*_*synth*,*dp*_ = 10. *D*, back to PFL 1∗ + MARCKS, with the parameters *k*_*unbind,c*_ and *k*_*inact*_ taken at 10× and 0.1× their base-case values, representing hypothetical *K*_*c*_ and *K*_*a*_ mutations, respectively. DAG, diacylglycerol; MARCKS, myristoylated alanine-rich C-kinase substrate; PA, phosphatidic acid; PFL, positive feedback loop; PLC, phospholipase C.

The combined model with PFL 1∗ was able to recapitulate two key findings from the analysis of the original model (12). First is the prediction concerning the critical effect of DAG kinase activity on the responsiveness of the system. The combined model showed that reducing the corresponding rate constant, *k*_DAGK_, by 0.3× dramatically diminishes polarization, whereas increasing it by three times enhances the degree of polarization (Fig. 7*A*). Analysis of the DAG and PA concentrations shows that the abundance of DAG at the back of the cell is reduced to a much greater extent than it is at the front when *k*_DAGK_ is increased (Fig. 7*B*). Second, in the prior work, another PFL was considered, in which active PKC promotes DAG production through activation of phospholipase D (PFL 2). When PFL 2 was added to the combined model with PFL 1∗ and regulation of MARCKS, the level of amplification was substantially increased, in line with the previously observed synergy among the feedback mechanisms (Fig. 7*C*).

Finally, using the combined model with PFL 1∗ and MARCKS regulation, we assessed the effects of hypothetical activating PLC mutations on polarization of the pathway. The *K*_*c*_ and *K*_*a*_ mutation mechanisms were introduced, respectively, by increasing *k*_*unbind,c*_ by 10× and by reducing *k*_*inact*_ by 0.1×. Noting that these parameter variations shifted the dose response to lower levels of mean fractional occupancy, we found that both these variants diminished the degree of polarization of the pathway, with the *K*_*a*_ mutation showing the more deleterious effect (Fig. 7*D*).

## Discussion

The refined structure of full-length PLC-γ1 has elucidated a more detailed mechanism of the enzyme’s activation (5, 6). The multistep process relies on the coordination and interaction of multiple PLC-γ1 domains, together with the phosphorylation of Tyr783, resulting in relief of basal autoinhibition. Activation of PLC-γ1 is important for chemotaxis, platelet aggregation, and adaptive immunity (2, 13, 14), and failure to regulate activation of PLC-γ1 has been implicated in emergence of immune disorders and certain cancers (3). To understand the mechanism of PLC-γ1 activation more quantitatively, we adopted a rule-based formalism to define differential equation models. We investigated how PLC-γ1 domain interactions and phosphorylation of Tyr783 affect activation of the enzyme and its function in cells, and the models are publicly available for others to use and build upon.

We first investigated the phosphorylation of Tyr783 and its role in releasing the autoinhibition of the enzyme. The phosphorylation itself is not expected to affect the autoinhibition; rather, it serves as a binding site for the cSH2 domain that is normally bound to a C2 domain blocking the catalytic core. When the cSH2 domain binds Tyr783, a structural rearrangement occurs that uncovers the core, allowing it to engage the membrane. Model analysis and simulations identified the conditions that must hold if favorable Tyr783 phosphorylation kinetics and higher affinity of the cSH2–pTyr783 interaction substantially increase PLC-γ1 activation. Most of the evidence indicates that Tyr783 phosphorylation is necessary for relieving the autoinhibition of PLC-γ1; however, a number of other studies have implicated other PLC-γ1 phosphorylation sites that might play a role in activation. Depending on the cell type, the receptor, and whether experiments were carried out *in vitro* or in whole cells, different tyrosine phosphorylation sites have been shown to enhance PLC-γ1 activity (15, 16, 17). In future work, variations of this model could be developed in conjunction with experiments to clarify the roles of those phosphorylation sites.

The model further predicted that various reaction rates differentially affect the maintenance of active PLC-γ1 species at the membrane. Unlike the steady-state magnitude of the enzymatic activity, its lifetime is relatively insensitive to changes in the Tyr783 phosphorylation rate constant. In contrast, the lifetime is positively influenced by the frequency of the catalytic core binding to the membrane, *k*_*act*_. We attribute this sensitivity to the two-step inactivation process, which requires PLC-γ1 to dissociate from both the membrane and the receptor before returning to the cytosol. Increasing the value of *k*_*act*_ allows the enzyme to more rapidly rebind the membrane while it remains bound to the receptor. Thus, PLC-γ1 can well at the membrane, unbinding and then rebinding the membrane or the receptor to prolong activation. We previously proposed that the lifetime of PLC-γ1 activity at the membrane might be enhanced, through a positive feedback *via* the enzyme’s reported interaction with PA at membranes (18), as a mechanism that polarizes downstream signaling to bias chemotactic cell migration (12). We modeled this as a decrease in the effective off-rate of PLC-γ1 from the receptor complex. In the more comprehensive model considered here, we modified the PFL, proposing that PA enhances association of the catalytic core with the membrane. The modified feedback loop, in conjunction with regulation of MARCKS, was able to polarize the pathway and bestowed improved stability of the polarized pattern relative to the previous model.

The structure-based model also shed light on how activating PLC-γ1 mutations associated with certain cancers and other diseases (5) might manifest in the dynamics of enzyme activation in cells. Two mechanisms that have been postulated to explain the function of these variants include disruptions of the autoinhibitory interaction, which we refer to as *K*_*c*_ mutations, and gains in PLC-γ1 membrane affinity, which we refer to as *K*_*a*_ mutations. Whereas activating mutations found in the autoinhibitory interfaces between the cSH2 domain and the catalytic core (such as P867R and D1165H) are good candidates for disrupting *K*_*c*_, others (such as R48W, R687W, and R753H) could contribute to disrupting *K*_*c*_, enhancing *K*_*a*_, or both (6). While both types of mutations increase steady-state enzyme activity, they are predicted to have distinct effects on the activation kinetics, relative to wildtype PLC-γ1; *K*_*c*_ mutations, like Tyr783 phosphorylation kinetics, primarily affect priming of the enzyme by relaxing the major barrier in the activation sequence, whereas *K*_*a*_ mutations primarily promote maintenance of the active state by effectively hindering the complete inactivation sequence. This qualitative difference is manifested in the kinetics of PLC-γ1 activation, which could inform the quantitative characterization of PLC-γ1 (and corresponding PLC-γ2) variants. Interestingly, both kinds of activating “mutations” diminished the ability to polarize PLC/PKC signaling in our combined model, consistent with previous analyses illustrating the need to suppress chemotactic signaling except at the leading edge (19, 20).

Despite the prevalence of PLC-γ1 (and the related PLC-γ2) mutations in various disease states, there are currently no specific pharmacological compounds available for the study or treatment of related diseases (3). Given the recent structural insights, together with dynamical insights derived from mathematical models, it seems possible that drugs will be developed to selectively disrupt PLC-γ1/2 activation and/or to limit the lifetime of active PLC-γ1/2 at the membrane. To advance the present modeling strategy, we envision that the model might be refined to include the influences of the PH and SH3 domains. Such refinement would increase the combinatorial complexity of the system, but perhaps it would allow us to investigate other mechanisms of disease. For example, the split PH domain in the X–Y linker is associated with maintaining the autoinhibition of the core. It binds to the hydrophobic ridge of the core while also interacting with the cSH2 domain (6). As a result, known mutations in the split PH domain could also contribute to the unregulated release of autoinhibition. The SH3 domain, also found in the X–Y linker, interacts with a number of signaling and adaptor proteins, which has made it a potential drug target for PLC-γ1 (21). When proteins bind the SH3 domain, they could influence autoinhibition or/and the dwell time of PLC-γ1 at the membrane. On the other hand, known mutations in the SH3 domain could alter those dynamics. We envision that a refined version of this model, supported by new experiments, will allow even greater insight to advance our understanding of normal and aberrant PLC-γ1/2 function in cells. And a similar strategy, to the extent justified by structural knowledge, may be used to model the dynamics of other enzymes that are regulated through receptor-mediated and membrane interactions.

## Experimental procedures

### Kinetic model of PLC-γ1 regulation and activation

The model specifies two molecules: an RTK, which is assumed to be in its active and phosphorylated state, with a phosphotyrosine site for enzyme recruitment, and the enzyme PLC-γ1. Components of the PLC-γ1 molecule include the nSH2 and cSH2 domains, the Tyr783 phosphorylation site, and the catalytic core. The autoinhibition of PLC-γ1 is modeled as reversible intramolecular binding of the cSH2 domain to the core, provided that the cSH2 is unbound and the core is inactive. PLC-γ1 engages active receptors *via* reversible binding of the nSH2 domain to the RTK phosphotyrosine site. While PLC-γ1 is bound to the RTK, Tyr783 can be phosphorylated by the RTK. Phosphorylated Tyr783 competes with the inactive core for the cSH2 domain, thus relieving the autoinhibition; while bound to cSH2, phosphorylated Tyr783 is protected from dephosphorylation (22, 23). Whenever the core is not occupied by cSH2, it can bind to the membrane and thus become active.

Using the interface with BioNetGen rule–based network generator (24) within the freely available Virtual Cell (VCell) software environment (10), the interactions and state transitions described previously were codified as a list of 12 rules and their associated rate constants; considering detailed balance, 11 of the rate constant values are independent (Table 1). From those rules, a network of 17 species and 53 reactions was generated (Fig. S1). For analysis of the kinetic model as a compartmental and deterministic system, 17 ordinary differential equations in time were solved using the Combined Stiff Solver (Implicit Differential Algebraic/CVODE) of VCell, with a maximum time step of 1 s. The solver was run for 5000 s to ensure that the steady state of the system was accurately estimated. This Biomodel and primary simulations are publicly available in VCell under user name jnosbis, Biomodel name ”Nosbisch PLCgamma1 2021.”

**Table 1:** Reaction rules and associated parameters of the PLC-γ1 model

Reaction rule	Rate constant	Base value
nSH2 binding to RTK when core is inactive	*k*_*on*___*nSH2*_	1 μM^−1^ s^−1^
nSH2 binding to RTK when core is active	*k*_*on_nSH2_2*_	χ*k*_*on_nSH2*_; χ = 1000
nSH2 unbinding RTK	*k*_*off_nSH2*_	1 s^−1^
Tyr783 phosphorylation	*k*_*phos*_	1 s^−1^
Tyr783 dephosphorylation	*k*_*dephos*_	1 s^−1^
cSH2 binding core	*k*_*bind,c*_	100 s^−1^
cSH2 unbinding core	*k*_*unbind,c*_	1 s^−1^
cSH2 binding Tyr783	*k*_*bind,p*_	100 s^−1^
cSH2 unbinding Tyr783	*k*_*unbind,p*_	1 s^−1^
Core activation with nSH2 unbound	*k*_*act*_	0.01 s^−1^
Core activation with nSH2 bound	*k*_*act*_2_	χ*k*_act_
Core inactivation	*k*_*inact*_	0.1 s^−1^

### Assignment of model parameter values

The initial concentration of PLC-γ1 was set to 0.02 μM to be consistent with previous modeling work (12). The concentration of the RTK (on a whole-cell basis) was considered the input to the model and was varied within the range of 0.005 to 0.05 μM. The association and dissociation rate constants for the nSH2–RTK interaction (*k*_*on_nSH2*_ = 1 μM^−1^ s^−1^ and *k*_*off_nSH2*_ = 1 s^−1^, respectively) yield a dissociation constant (*K*_*D*_) of 1 μM, consistent with the reported ranges for monovalent SH2 domain interactions with phosphotyrosines (25, 26). When PLC-γ1 is in the active state, it is bound by membrane lipids, and therefore, the binding of nSH2 and RTK is enhanced by induced proximity at the plasma membrane; to account for this, the associated rate constant, *k*_*on_nSH2_2*_, is related to *k*_*on_nSH2*_ by a factor χ = 1000 (27). The Tyr783 phosphorylation and dephosphorylation rate constants (*k*_*phos*_ and *k*_*dephos*_, respectively) were systematically varied, with base values of 1 s^−1^ for both. The frequencies of cSH2 domain binding and unbinding the core (*k*_bind,c_ = 100 s^−1^ and *k*_*unbind,c*_ = 1 s^−1^, respectively) represent rapid intramolecular transitions that favor the bound (autoinhibited) state when the RTK is absent. The frequencies of cSH2 domain binding and unbinding phosphorylated Tyr783 (*k*_*bind,p*_ and *k*_*unbind,p*_, respectively) were assigned the same base values as for the cSH2–core interaction. The frequency of PLC-γ1 activation (when the core is available to bind membrane) depends on whether the enzyme is cytosolic (*k*_*act*_ = 0.01 s^−1^) or in complex with the RTK *via* nSH2 (*k*_*act*_*_*_*2*_ = χ*k*_act_ = 10 s^−1^). These values yield fast membrane binding when PLC-γ1 is bound to the RTK and slow binding when the enzyme is being activated directly from the cytosol. The use of the same enhancement factor χ satisfies detailed balance for the assembly of the canonically active state, in which the nSH2 is bound to RTK and the core is bound to membrane. Finally, the frequency of PLC-γ1 inactivation (*k*_*inact*_ = 0.1 s^−1^) implies that the active state, once achieved, is longer lived than the other dynamic states of PLC-γ1.

### Integrating structure-based PLC-γ1 activation in a model of PLC/PKC polarization

The new PLC-γ1 activation model was combined with a published model of PLC/PKC polarization (12). To do this, the reaction term describing (concomitant) PLC-γ1 recruitment and activation in the latter (*V*_*PLC*_) was replaced with the present reaction network; thus, the polarization model was increased in complexity, from only two PLC-γ1 states (inactive/cytosolic and active/receptor bound) to 16 PLC-γ1 states. To integrate these species in the rate laws of the polarization model, three adjustments were made. First, in the rate laws for binding of the cytosolic PLC-γ1 species with available active receptors, the local density of the latter, (*r* – *e*) (12) was replaced with the density of active receptors, *r*, minus the sum of the eight RTK-bound PLC-γ1 species. Second, in the rate law for PLC-catalyzed PIP_2_ hydrolysis (*V*_*hyd,PLC*_), active PLC was evaluated as the sum of the six active PLC-γ1 species. Third, in the stabilization of PLC-γ1 recruitment by PA (model variable *d*_*p*_), referred to as PFL 1 (12), the effect is envisioned to enhance the frequency of PLC-γ1 binding to membrane lipids (*k*_*act*_); the rate constants *k*_*act*_ and *k*_*act_2*_ in the structure-based model are each modified by the factor $\left(1+K_{PA}d_{p}\right)$, where the constant *K*_*PA*_ = 10 μm^2^ is defined in a manner consistent with, and has the same value as, the corresponding parameter (12).

Consistent with the polarization model, the molecular diffusivities of all cytosolic PLC-γ1 species were set at 19 μm^2^/s, and the diffusivities for all receptor- and/or membrane-bound PLC-γ1 species were set at 0.01 μm^2^/s. All other initial concentrations, molecular diffusivities, and rate constants were the same as the base-case values in the polarization model (12). This includes the on-rates and off-rates for PLC binding to the receptor, with *k*_*on_nSH2*_ = 0.1 μM^−1^ s^−1^ and *k*_*on_nSH2*_ = 0.1 s^−1^, respectively. All other rate constants of the structure-based model were set to the base-case values in Table 1. This Biomodel and primary simulations are publicly available in VCell under user name jnosbis, Biomodel name “Nosbisch PLCgamma1 + polarization 2021.”

## Data availability

The model and primary simulations associated with main Figure 2, Figure 3, Figure 4, Figure 5, S2 and S3 of the supporting information are publicly available in the Virtual Cell software environment (www.vcell.org) under user name jnosbis, Biomodel name “Nosbisch PLCgamma1 2021.” For the model and primary simulations associated with Figures 6 and 7, the Biomodel name is “Nosbisch PLCgamma1 + polarization 2021.”

## Supporting information

This article contains supporting information.

## Conflict of interest

The authors declare that they have no conflicts of interest with the contents of this article.
