# Supplementary material for: A kinetic model of phospholipase C-γ1 linking structure-based insights to dynamics of enzyme autoinhibition and activation
Source: J Biol Chem. 2022 Mar 31;298(5):101886. doi: 10.1016/j.jbc.2022.101886 (PMC9097458; doi:10.1016/j.jbc.2022.101886)
Supplement: Supplemental Text S1 [file mmc1.pdf]

Supplementary Text S1: Analysis of limiting cases

Notation used in this document

States of PLC-γ1:

We define the fraction of PLC-γ1 in a particular state as

P(f/b, u/p/b, f/b/a).

The first index refers to the state of the nSH2 domain (free/bound), the second index refers to the state of Tyr783 (unphosphorylated/phosphorylated/bound by cSH2), and the third index refers to the state of the catalytic core (free/'bound' by cSH2/active). For example, under unstimulated conditions, the most abundant state of wild-type PLC-γ1 is *P*(f,u,b). In that state, the nSH2 domain is free, Tyr783 is unphosphorylated, and the catalytic core is bound (occupied by cSH2).

Dimensionless equilibrium constants:

K_n: Equilibrium constant, nSH2/receptor (includes the free, active receptor density)

K_c: Equilibrium constant, cSH2/catalytic core

K_p: Equilibrium constant, cSH2/pTyr783

K_a: Equilibrium constant, catalytic core/membrane (PLC activation)

Except where noted otherwise, other parameters are as defined in the main text.

Limiting Case 1: Non-phophorylatable PLC-γ1 variant

Non-receptor-bound states:

$P(\text{f,u,b}) = K_c * P(\text{f,u,f})$

$P(\text{f,u,a}) = K_a * P(\text{f,u,f})$

Receptor-bound states:

$P(\text{b,u,f}) = K_n * P(\text{f,u,f})$

$P(\text{b,u,a}) = \chi * K_a * P(\text{b,u,f}) = \chi * K_n * P(\text{f,u,a}) = \chi * K_n * K_a * P(\text{f,u,f})$

$P(\text{b,u,b}) = K_n * P(\text{f,u,b}) = K_n * K_c * P(\text{f,u,f})$

Note the use of the parameter χ from the full model, which characterizes the dramatic enhancement of interactions when PLC-γ1 is membrane-associated, relative to when PLC-γ1 is cytosolic. From these equilibrium expressions and overall conservation of PLC-γ1 ($\sum P(*,*,*) = 1$), the fractions of PLC-γ1 in the various states are derived. The active fraction is

$$P(*,*,a) = \frac{K_a(1+\chi K_n)}{(1+K_n)(1+K_c)+K_a(1+\chi K_n)}.$$

This equation may be expressed as follows:

$$P(*,*,a) = \frac{A}{1+A};\ A = \frac{K_a(1+\chi K_n)}{(1+K_n)(1+K_c)},$$

where *A* is the ratio of active/inactive PLC.

Limiting Case 2: 'Phospho-mimetic' PLC-γ1 variant

Non-receptor-bound states:

P(f,p,b) = K_c*P(f,p,f)
P(f,p,a) = K_a*P(f,p,f)
P(f,b,f) = K_p*P(f,p,f)
P(f,b,a) = K_a*P(f,b,f) = K_p*K_a*P(f,p,f)

Receptor-bound states:

P(b,p,f) = K_n*P(f,p,f)
P(b,p,b) = K_c*P(b,p,f) = K_n*K_c*P(f,p,f)
P(b,p,a) = χ*K_a*P(b,p,f) = χ*K_n*P(f,p,a) = χ*K_n*K_a*P(f,p,f)
P(b,b,f) = K_n*P(f,b,f) = K_n*K_p*P(f,p,f)
P(b,b,a) = χ*K_n*P(f,b,a) = χ*K_a*P(b,b,f) = K_p*P(b,p,a) = χ*K_n*K_p*K_a*P(f,p,f)

We note that the effect of constitutive phosphorylation is negated by eliminating the interaction between pTyr783 and cSH2 (K_p = 0). Thus, this parameter substitution recapitulates Limiting Case 1.

As before, manipulation of the equilibrium equations above yields an expression for the fraction of PLC-γ1 in the active states, $P(*,*,a)$.

$$P(*,*,a) = \frac{K_a(1+\chi K_n)(1+K_p)}{(1+K_n)(1+K_c+K_p)+K_a(1+\chi K_n)(1+K_p)} = \frac{A}{1+A};$$

$$A = K_a\left(\frac{1+K_p}{1+K_c+K_p}\right)\left(\frac{1+\chi K_n}{1+K_n}\right).$$

The following ratio compares the scenario with versus without phosphorylation.

$$\frac{A}{A(K_p=0)} = \frac{(1+K_p)(1+K_c)}{1+K_c+K_p} = 1 + \frac{K_pK_c}{1+K_c+K_p}.$$

Therefore, its value must be >> 1, or

$$K_pK_c \gg 1+K_c+K_p,$$

if phosphorylation of PLC-γ1 has a dramatic effect on enzyme activation.

This simple analysis shows that if PLC-γ1 phosphorylation dramatically affects activation, K_p and K_c must both be >> 1; however, it is not necessary for K_p to be much larger than K_c.

Limiting Case 3: Pseudo-equilibrium kinetic scheme

Based on the above development, and with the express goal of developing intuition about the full network, we propose a kinetic scheme with the simplifying assumption that the states of the cSH2 domain are close to equilibrium. We recognize that this assumption is not generally valid and depends on the values of the rate constants in the full model. Neither Limiting Case 3 nor the subsequent Limiting Case 4 were used to calculate results shown in the paper.

With the assumption in place, we are able to lump the free and bound states of the catalytic core into an inactive state, $P(*,*,i) = P(*,*,f) + P(*,*,b)$, and to lump the phosphorylated and bound states of Tyr783 into a total phosphorylated state, $P(*,\hat{p},*) = P(*,p,*) + P(*,b,*)$. Conservation equations with interpretations are given below.

Receptor-bound, unphosphorylated, inactive:

$$\frac{dP(b,u,i)}{dt} \approx k'_{on\_nSH2}P(f,u,i) + \left(\frac{1+K_c}{1+K_c+K_p}\right)k_{dephos}P(b,\hat{p},i) + k_{inact}P(b,u,a) - \left(k_{off\_nSH2} + k_{phos} + \frac{\chi k_{act}}{1+K_c}\right)P(b,u,i)$$

This is the first receptor-bound PLC species formed after stimulation; note that, like the affinity constant K_n, the association frequency k'_{on_nSH2} includes the free receptor density (signified by the prime). $P(b,u,i)$ is readily phosphorylated with frequency k_{phos}, but it is not readily activated because of autoinhibition, which reduces the activation rate by the factor $1/(1+K_c)$.

Receptor-bound, phosphorylated, inactive:

$$\frac{dP(b,\hat{p},i)}{dt} \approx k'_{on\_nSH2}P(f,\hat{p},i) + k_{phos}P(b,u,i) + k_{inact}P(b,\hat{p},a) - \left[k_{off\_nSH2} + \left(\frac{1+K_c}{1+K_c+K_p}\right)k_{dephos} + \left(\frac{1+K_p}{1+K_c+K_p}\right)\chi k_{act}\right]P(b,\hat{p},i)$$

This is the first phosphorylated PLC species formed after stimulation. It is not dephosphorylated at the maximum rate, because the interaction of pTyr783 with the cSH2 domain (affinity constant K_p) offers some protection; however, that effect is at best modest when autoinhibition is strong ($K_c \sim K_p$ or larger). Activation of $P(b,\hat{p},i)$ is also tempered by autoinhibition, but occupation of the cSH2 domain by pTyr783 can enhance activation dramatically relative to $P(b,u,i)$.

Receptor-bound, phosphorylated, active:

$$\frac{dP(b,\hat{p},a)}{dt} \approx \chi k'_{on\_nSH2}P(f,\hat{p},a) + k_{phos}P(b,u,a) + \left(\frac{1+K_p}{1+K_c+K_p}\right)\chi k_{act}P(b,\hat{p},i) - \left(k_{off\_nSH2} + \frac{k_{dephos}}{1+K_p} + k_{inact}\right)P(b,\hat{p},a)$$

This is the 'canonical' active PLC species, thought to form predominantly by the sequence $P(f,u,i) \rightarrow P(b,u,i) \rightarrow P(b,\hat{p},i) \rightarrow P(b,\hat{p},a)$. While in this state, pTyr783 is not readily dephosphorylated, as the protection by cSH2 binding reduces the dephosphorylation frequency by the factor $1/(1+K_p)$. The nSH2 interaction and the interaction of the catalytic core with membrane readily dissociate, but they can rapidly reform at the rates preceded by the enhancement factor, χ.

Receptor-bound, unphosphorylated, active:

$$\frac{dP(b,u,a)}{dt} \approx \chi k'_{on\_nSH2} P(f,u,a) + \frac{k_{dephos}}{1+K_p} P(b,\hat{p},a) + \frac{\chi k_{act}}{1+K_c} P(b,u,i) - \left(k_{off\_nSH2} + k_{phos} + k_{inact}\right) P(b,u,a)$$

This is thought to be a minor intermediate species, as its formation pathways are unfavorable.

Cytosolic, unphosphorylated, inactive:

$$\frac{dP(f,u,i)}{dt} \approx k_{off\_nSH2} P(b,u,i) + k_{dephos}\left(\frac{1+K_c}{1+K_c+K_p}\right) P(f,\hat{p},i) + k_{inact} P(f,u,a) - \left(k'_{on\_nSH2} + \frac{k_{act}}{1+K_c}\right) P(f,u,i)$$

This is the predominant species in unstimulated cells and thought to be the major cytosolic species under all conditions.

Cytosolic, phosphorylated, inactive:

$$\frac{dP(f,\hat{p},i)}{dt} \approx k_{off\_nSH2} P(b,\hat{p},i) + k_{inact} P(f,\hat{p},a) - \left[k'_{on\_nSH2} + \left(\frac{1+K_c}{1+K_c+K_p}\right) k_{dephos} + \left(\frac{1+K_p}{1+K_c+K_p}\right) k_{act}\right] P(f,\hat{p},i)$$

This species is formed by full dissociation of phosphorylated PLC from the receptor and membrane interactions. Like $P(b,\hat{p},i)$, it is readily dephosphorylated; the dephosphorylation rate, relative to that of reestablishing the nSH2-receptor interaction, determines how rare or prominent this state is relative to $P(f,u,i)$.

Not receptor-bound, phosphorylated, active:

$$\frac{dP(f,\hat{p},a)}{dt} \approx k_{off\_nSH2} P(b,\hat{p},a) + k_{act}\left(\frac{1+K_p}{1+K_c+K_p}\right) P(f,\hat{p},i) - \left(\chi k'_{on\_nSH2} + \frac{k_{dephos}}{1+K_p} + k_{inact}\right) P(f,\hat{p},a)$$

This intermediate is predominantly formed from $P(b,\hat{p},a)$ by dissociation of nSH2 from receptor. It tends to be short-lived, because nSH2 can rapidly rebind while PLC remains at the membrane.

Not receptor-bound, unphosphorylated, active:

$$\frac{dP(f,u,a)}{dt} \approx k_{off\_nSH2} P(b,u,a) + \frac{k_{dephos}}{1+K_p} P(f,\hat{p},a) + \frac{k_{act}}{1+K_c} P(f,u,i) - \left(\chi k'_{on\_nSH2} + k_{inact}\right) P(f,u,a)$$

Like $P(b,u,a)$, this is thought to be a minor intermediate species, as its formation pathways are unfavorable.

Limiting Case 4: Attempt at a 'compact' steady-state expression

The scheme above, though illustrative, is still too complex for derivation of compact, steady-state expressions. Further simplification was achieved by assuming that $P(b,u,a)$ and $P(f,u,a)$ and the reactions that form them are negligible. <u>We recognize that this assumption, which essentially makes PLC-γ1 phosphorylation a requirement for activation, is restrictive and not generally applicable</u>. We offer this derivation to further develop understanding of the reaction network and to demonstrate its complexity even after considerable simplification. The following steady-state equations, formulated with the aforementioned assumptions, are presented in the order by which the final expression was derived.

Cytosolic, phosphorylated, inactive:

$$P(f,\hat{p},i) \approx \frac{k_{off\_nSH2}P(b,\hat{p},i) + k_{inact}P(f,\hat{p},a)}{k'_{on\_nSH2} + \left(\frac{1+K_c}{1+K_c+K_p}\right)k_{dephos} + \left(\frac{1+K_p}{1+K_c+K_p}\right)k_{act}}$$
$$= \frac{k_{off\_nSH2}P(b,\hat{p},i) + k_{inact}P(f,\hat{p},a)}{k_{sum}};$$
$$k_{sum} = k'_{on\_nSH2} + \left(\frac{1+K_c}{1+K_c+K_p}\right)k_{dephos} + \left(\frac{1+K_p}{1+K_c+K_p}\right)k_{act}.$$

We further define, as a means of shorthand,

$$\phi_{on} = \frac{k'_{on\_nSH2}}{k_{sum}}; \quad \phi_{act} = \left(\frac{1+K_p}{1+K_c+K_p}\right)\frac{k_{act}}{k_{sum}}.$$

Not receptor-bound, phosphorylated, active:

$$P(f,\hat{p},a) \approx \frac{k_{off\_nSH2}P(b,\hat{p},a) + k_{act}\left(\frac{1+K_p}{1+K_c+K_p}\right)P(f,\hat{p},i)}{\chi k'_{on\_nSH2} + k_{inact}}$$
$$\approx \frac{k_{off\_nSH2}[P(b,\hat{p},a) + \phi_{act}P(b,\hat{p},i)]}{\chi k'_{on\_nSH2} + (1-\phi_{act})k_{inact}}$$

Plug this result into $P(f,\hat{p},i)$ and simplify, incorporating the nSH2 affinity constant, $K_n = k'_{on\_nSH2}/k_{off\_nSH2}$:

$$P(f,\hat{p},i) \approx \frac{\phi_{on}}{K_n}\left[\frac{(\chi k'_{on\_nSH2} + k_{inact})P(b,\hat{p},i) + k_{inact}P(b,\hat{p},a)}{\chi k'_{on\_nSH2} + (1-\phi_{act})k_{inact}}\right]$$

Receptor-bound, phosphorylated, active:

$$P(b,\hat{p},a) \approx \frac{\chi k'_{on\_nSH2}P(f,\hat{p},a) + \left(\frac{1+K_p}{1+K_c+K_p}\right)\chi k_{act}P(b,\hat{p},i)}{k_{off\_nSH2} + k_{inact}}$$
$$\approx a\left(\frac{1+K_p}{1+K_c+K_p}\right)\chi K_a P(b,\hat{p},i);$$

$$a = \frac{\chi k'_{on\_nSH2} + \phi_{on} k_{off\_nSH2} + (1 - \phi_{act}) k_{inact}}{\chi k'_{on\_nSH2} + (1 - \phi_{act})\left(k_{off\_nSH2} + k_{inact}\right)}$$

Once again, plug the result into $P(f, \hat{p}, i)$ and simplify further:

$$P(f, \hat{p}, i) \approx \frac{b}{K_n} P(b, \hat{p}, i);$$

$$b = \phi_{on} \left[\frac{\chi k'_{on\_nSH2} + k_{inact} + a \left(\frac{1 + K_p}{1 + K_c + K_p} \right) \chi k_{act}}{\chi k'_{on\_nSH2} + (1 - \phi_{act}) k_{inact}} \right]$$

$$= \frac{(\phi_{on} + a \phi_{act}) \chi k'_{on\_nSH2} + \phi_{on} k_{inact}}{\chi k'_{on\_nSH2} + (1 - \phi_{act}) k_{inact}}$$

Receptor-bound, phosphorylated, inactive:

$$P(b, \hat{p}, i) \approx \frac{k_{phos} P(b, u, i) + k'_{on\_nSH2} P(f, \hat{p}, i) + k_{inact} P(b, \hat{p}, a)}{k_{off\_nSH2} + \left(\frac{1 + K_c}{1 + K_c + K_p} \right) k_{dephos} + \left(\frac{1 + K_p}{1 + K_c + K_p} \right) \chi k_{act}} \approx c P(b, u, i);$$

$$c = \frac{k_{phos}}{(1 - b) k_{off\_nSH2} + \left(\frac{1 + K_c}{1 + K_c + K_p} \right) k_{dephos} + (1 - a) \left(\frac{1 + K_p}{1 + K_c + K_p} \right) \chi k_{act}};$$

Receptor-bound, unphosphorylated, inactive:

$$P(b, u, i) \approx \frac{k'_{on\_nSH2} P(f, u, i) + \left(\frac{1 + K_c}{1 + K_c + K_p} \right) k_{dephos} P(b, \hat{p}, i)}{k_{off\_nSH2} + k_{phos}} \approx d K_n P(f, u, i);$$

$$d = \frac{k_{off\_nSH2}}{k_{off\_nSH2} + k_{phos} \left[\frac{(1 - b) k_{off\_nSH2} + (1 - a) \left(\frac{1 + K_p}{1 + K_c + K_p} \right) \chi k_{act}}{(1 - b) k_{off\_nSH2} + \left(\frac{1 + K_c}{1 + K_c + K_p} \right) k_{dephos} + (1 - a) \left(\frac{1 + K_p}{1 + K_c + K_p} \right) \chi k_{act}} \right]}$$

$$= \frac{k_{off\_nSH2} + \gamma \left(\frac{1 + K_c}{1 + K_c + K_p} \right) k_{dephos}}{k_{phos} + k_{off\_nSH2} + \gamma \left(\frac{1 + K_c}{1 + K_c + K_p} \right) k_{dephos}};$$

$$\gamma = \frac{k_{off\_nSH2}}{(1 - b) k_{off\_nSH2} + (1 - a) \left(\frac{1 + K_p}{1 + K_c + K_p} \right) \chi k_{act}}$$

$$= \frac{1}{1 - b + \left(\frac{1 + K_p}{1 + K_c + K_p} \right) \left[\frac{(1 - \phi_{act} - \phi_{on}) \chi k_{act}}{\chi k'_{on\_nSH2} + (1 - \phi_{act})\left(k_{off\_nSH2} + k_{inact}\right)} \right]}$$

Derivation of the active fraction:

At this point, all of the states may be expressed in terms of the major cytosolic state, $P(f, u, i)$.

The strategy now is to assemble the active and inactive fractions [$P(*,*,a)$ and $P(*,*,i)$, respectively] in terms of $P(f,u,i)$, then take their ratio (previously defined as the quantity A) to eliminate $P(f,u,i)$.

$$P(*,*,a) \approx P(b,\hat{p},a) + P(f,\hat{p},a)$$

$$\approx \left[a\left(\frac{1+K_p}{1+K_c+K_p}\right)\chi K_a\left(1+\frac{k_{off\_nSH2}}{\chi k'_{on\_nSH2}+(1-\phi_{act})k_{inact}}\right) + \frac{\phi_{act}k_{off\_nSH2}}{\chi k'_{on\_nSH2}+(1-\phi_{act})k_{inact}}\right]P(b,\hat{p},i)$$

$$\approx \left(\frac{1+K_p}{1+K_c+K_p}\right)K_a\left(a\chi K_n + \frac{a\chi k'_{on\_nSH2}+\phi_{on}k_{inact}}{\chi k'_{on\_nSH2}+(1-\phi_{act})k_{inact}}\right)cdP(f,u,i);$$

$$P(*,*,i) \approx P(f,u,i) + P(f,\hat{p},i) + P(b,u,i) + P(b,\hat{p},i) \approx [1+bcd+(1+c)dK_n]P(f,u,i)$$

Evaluating the ratio, $A = P(*,*,a)/P(*,*,i)$,

$$A \approx \left(\frac{1+K_p}{1+K_c+K_p}\right)K_a\left(a\chi K_n + \frac{a\chi k'_{on\_nSH2}+\phi_{on}k_{inact}}{\chi k'_{on\_nSH2}+(1-\phi_{act})k_{inact}}\right)\frac{cd}{1+bcd+(1+c)dK_n},$$

with the quantity cd simplifying to

$$cd = \frac{\gamma k_{phos}}{k_{phos}+k_{off\_nSH2}+\gamma\left(\frac{1+K_c}{1+K_c+K_p}\right)k_{dephos}}.$$

Comparison to the "phosphomimetic," Limiting Case 2:

Exact agreement with the Limiting Case 2 result was found by setting $k_{phos} \to \infty, k_{dephos} \approx 0$. With those substitutions,

$$\phi_{on}+\phi_{act} \approx 1;\;\; a \approx 1;\;\; b \approx 1;\;\; d \approx \frac{k_{off\_nSH2}}{k_{phos}} \ll 1;\;\; cd \approx \gamma \approx \frac{1}{1-b} \gg 1;$$

$$A \approx \left(\frac{1+K_p}{1+K_c+K_p}\right)K_a\left(\frac{1+\chi K_n}{1+K_n}\right).$$

Rapid dephosphorylation in the cytosol:

After phosphorylated PLC-γ1 dissociates completely and returns to the cytosol, it is rapidly dephosphorylated if ϕ_{on} and ϕ_{act} are << 1; this is thought to be the case for wild-type PLC-γ1. If so, the probability of dephosphorylation, $1-\phi_{act}-\phi_{on}$, would be approximately unity, and,

$$a \approx \frac{\chi k'_{on_{nSH2}}+k_{inact}}{\chi k'_{on_{nSH2}}+k_{off,n}+k_{inact}};\;\; b \ll 1;$$

$$\gamma \approx \frac{\chi k'_{on\_nSH2}+k_{off\_nSH2}+k_{inact}}{\chi k'_{on\_nSH2}+k_{off\_nSH2}+k_{inact}+\left(\frac{1+K_p}{1+K_c+K_p}\right)\chi k_{act}}.$$

The result for the quantity γ is referred to below.

Sensitivity of PLC-γ1 activation to phosphorylation/dephosphorylation rates:

The expression for the active fraction suggests that the efficiency of PLC-γ1 activation relative to the frequency of phosphorylation, k_{phos}, is determined by the quantity,

$$\frac{k_{phos}}{k_{phos} + k_{off\_nSH2} + \gamma\left(\frac{1 + K_c}{1 + K_c + K_p}\right)k_{dephos}}.$$

As one might intuit, this efficiency factor compares k_{phos} to the frequency of dissociation, k_{off_nSH2} (PLC-γ1 must remain bound to an active receptor long enough to be phosphorylated), and to the frequency of dephosphorylation once the enzyme is phosphorylated. The dephosphorylation rate constant, k_{dephos}, is modified by the factor $(1 + K_c)/(1 + K_c + K_p)$, the fraction of the time pTyr783 is not protected by cSH2 while in a $P(*, \hat{p}, i)$ state (PLC-γ1 is phosphorylated, but the catalytic core is not fully engaged with the membrane). Another quantity that appears in the dephosphorylation term is the factor γ, its compactness belying the complexity of the numerous rate constants it encapsulates (see the expression in the previous subsection, for example). Those rate constants influence the protection of pTyr783 from dephosphorylation while phosphorylated PLC-γ1 shuttles between inactive and active states at the plasma membrane [$P(b, \hat{p}, i) \leftrightharpoons P(b, \hat{p}, a) \leftrightharpoons P(f, \hat{p}, a)$]. As the activation rate constant k_{act} is increased, γ is reduced; dephosphorylation is progressively disfavored. Based on the development of Limiting Case 3, it is understood that this is because active species are defined by a state of the catalytic core that is not available to bind the cSH2 domain, which then tends to bind and protect pTyr783.
