# Supplementary material for: A kinetic model of phospholipase C-γ1 linking structure-based insights to dynamics of enzyme autoinhibition and activation
Source: J Biol Chem. 2022 Mar 31;298(5):101886. doi: 10.1016/j.jbc.2022.101886 (PMC9097458; doi:10.1016/j.jbc.2022.101886)
Supplement: Supplemental Figures S1–S3 [file mmc2.pdf]

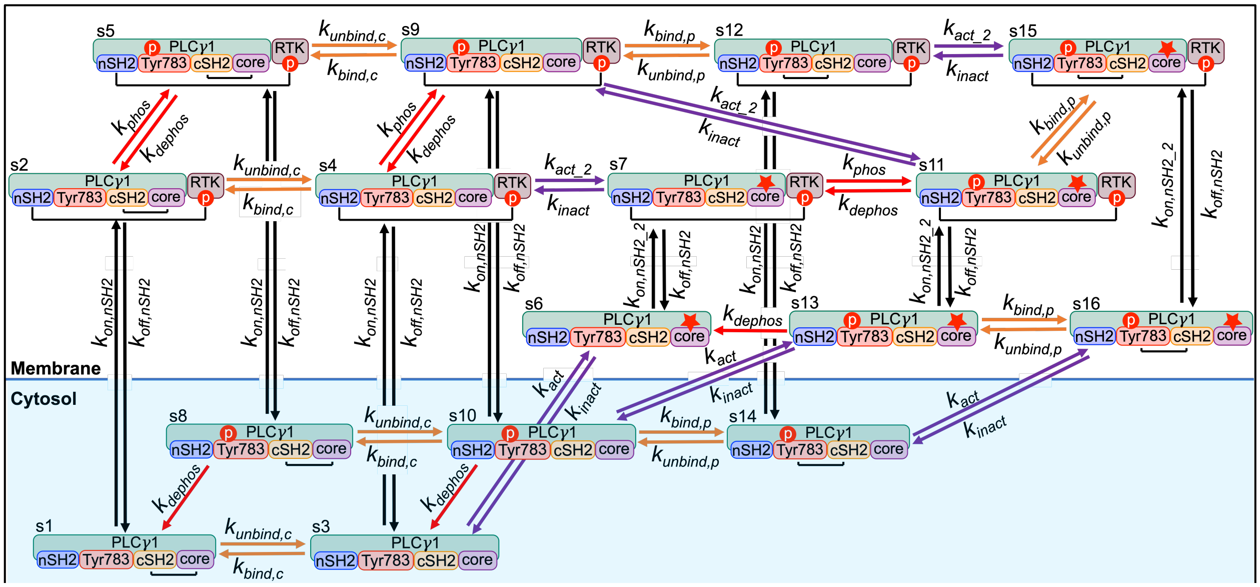

Figure S1: Full network diagram of the PLC-γ1 kinetic model. Application of the 12 reaction rules (Table 1) generated 17 species, labeled s0 – s16 by BioNetGen/VCell. The diagram depicts s1 – s16, the states of PLC-γ1; s0, RTK with free phosphotyrosine, is not shown. The cytosolic and membrane-proximal PLC-γ1 states are demarcated; the latter are receptor-bound (depicted by RTK in the complex), membrane-bound/active (depicted by a red asterisk above the core component), or both. The 12 rules correspond to 12 rate constants (considering detailed balance, 11 of them are independent), but due to the combinatorial manner by which the rules are applied, there are 53 distinct reactions.

A

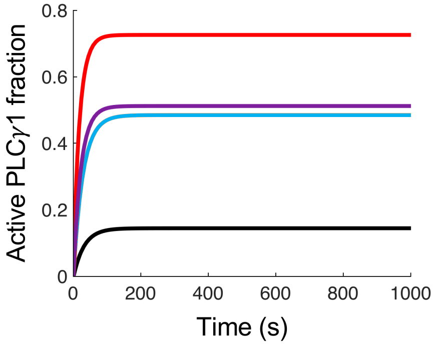

Parameters	$t_{1/2}$ (s)
$k_{bind,c}$ = 100 s^{-1}; $k_{unbind,c}$ = 1 s^{-1}	22
$k_{bind,c}$ = 10 s^{-1}; $k_{unbind,c}$ = 1 s^{-1}	20
$k_{bind,c}$ = 100 s^{-1}; $k_{unbind,c}$ = 10 s^{-1}	16
$k_{bind,c}$ = 10 s^{-1}; $k_{unbind,c}$ = 10 s^{-1}	12

B

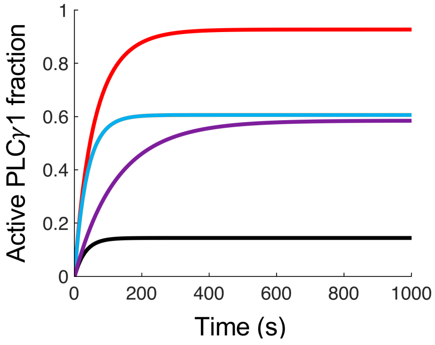

Parameters	$t_{1/2}$ (s)
k_{act} = 0.01 s^{-1}; k_{inact} = 0.1 s^{-1}	22
k_{act} = 0.1 s^{-1}; k_{inact} = 0.1 s^{-1}	26
k_{act} = 0.01 s^{-1}; k_{inact} = 0.01 s^{-1}	88
k_{act} = 0.1 s^{-1}; k_{inact} = 0.01 s^{-1}	41

Figure S2: Predicted effects of hypothetical mutations on PLC-γ1 activation kinetics, with K_p = 10. The analysis shown in Fig. 5C&D was repeated with the rate constant $k_{bind,p}$ reduced from 100 s^{-1} to 10 s^{-1}, a ten-fold reduction in affinity. (A) Time courses of PLC-γ1 activation comparing wild-type parameter values (black) to various K_c mutation scenarios. The corresponding $t_{1/2}$ values are tabulated. (B) Same as A, except for K_a mutation scenarios.

A

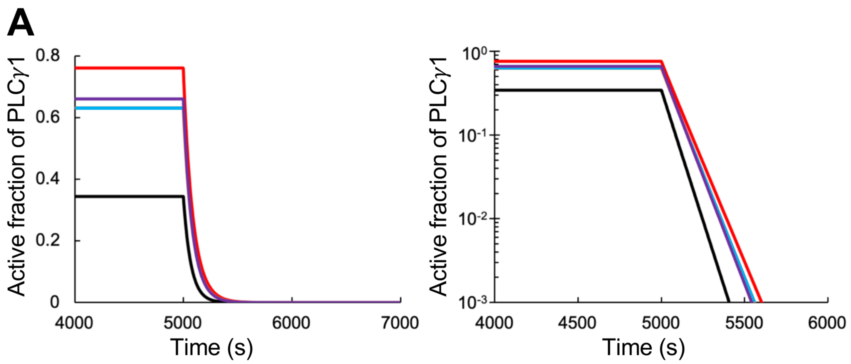

Parameters	$t_{1/2}$ (s)
$k_{bind,c}$ = 100 s^{-1}; $k_{unbind,c}$ = 1 s^{-1}	49
$k_{bind,c}$ = 10 s^{-1}; $k_{unbind,c}$ = 1 s^{-1}	59
$k_{bind,c}$ = 100 s^{-1}; $k_{unbind,c}$ = 10 s^{-1}	57
$k_{bind,c}$ = 10 s^{-1}; $k_{unbind,c}$ = 10 s^{-1}	61

B

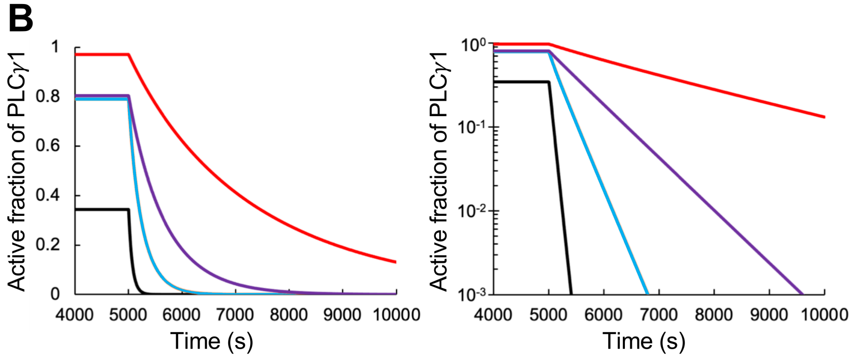

Parameters	$t_{1/2}$ (s)
k_{act} = 0.01 s^{-1}; k_{inact} = 0.1 s^{-1}	49
k_{act} = 0.1 s^{-1}; k_{inact} = 0.1 s^{-1}	167
k_{act} = 0.01 s^{-1}; k_{inact} = 0.01 s^{-1}	464
k_{act} = 0.1 s^{-1}; k_{inact} = 0.01 s^{-1}	1598

C

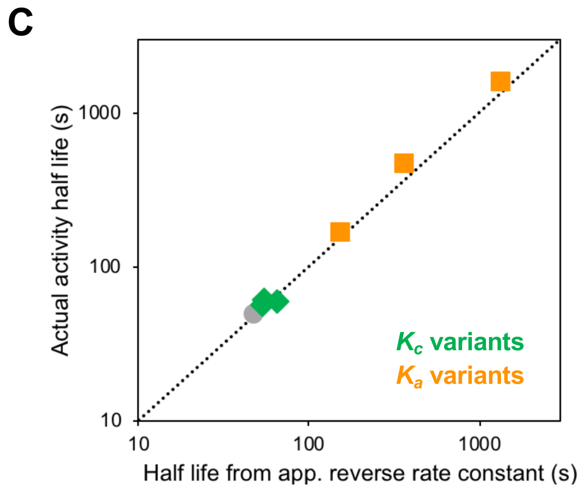

Figure S3: Lifetime analysis of hypothetical PLC-γ1 variants. For the hypothetical mutations analyzed in Figs. 4 & 5 of the main paper, lifetime analysis was performed as presented in Fig. 3 of the main paper: (A) K_c variants; (B) K_a variants. In both cases, the decay kinetics for the wild-type (base-case) parameter values (black) are shown for comparison. (C) For each variant and the wild-type, the activity half life from the lifetime analysis ($t_{1/2}$) is compared to a value estimated from the apparent reverse rate constant, according to $t_{1/2}\ (\text{estimated}) = \frac{\ln 2}{k_{r,app}}$.
